# Chemical Toxicants Used for Food Preservation in Africa. Is it a Case of Ignorance or Food Fraud? A Review

**DOI:** 10.1002/hsr2.70333

**Published:** 2025-04-18

**Authors:** Nnabueze Darlington Nnaji, Helen Onyeaka, Kingsley Tochukwu Ughamba, Chukwuebuka Maxwell Ononugbo, Chinasa Valerie Olovo, Ifeanyi Michael Mazi

**Affiliations:** ^1^ School of Chemical Engineering University of Birmingham Birmingham UK; ^2^ Department of Microbiology University of Nigeria Nsukka Nigeria; ^3^ Department of Science Laboratory Technology University of Nigeria Nsukka Nigeria; ^4^ Department of Biotechnology, Graduate School of Engineering Osaka University Yamadaoka Japan; ^5^ Department of Biochemistry and Molecular Biology, School of Medicine Jiangsu University Zhenjiang Jiangsu China; ^6^ Department of Microbiology University of Benin Benin City Nigeria

**Keywords:** Africa, chemical preservatives, chemical toxicants, food, food fraud, food preservation, preservatives

## Abstract

**Background and Aims:**

This review examined the use of chemical preservatives in food preservation and the food fraud risks associated with its misuse in Africa. In Africa, there is growing reliance on the use of synthetic chemical preservation such as benzoates, nitrates or sorbates to preserve foods to improve the storage life of the foods, however, the consumers are the eventual victims of the devastating health consequences associated with the use of these preservatives. The review also delves into the issue of food fraud, which involves the intentional addition of harmful chemicals to food products with the aim to prolong their shelf life or improve their look, ultimately resulting in detrimental health effects.

**Methods:**

This study employs a comprehensive methodology involving a literature review, data collection from diverse sources, case study analysis, technological assessment of food fraud detection, and policy review to examine the use of chemical toxicants in food preservation and their regulation across Africa.

**Results:**

The risks of chemical adulteration are demonstrated by case studies from several African nations, including the use of formalin to preserve meat and hazardous chemicals in bread and dairy products and the use of chemicals like dichloro‐diphenyl‐trichloroethane, sniper, and gamalin‐20 in preserving fruits and vegetables. The review also emphasizes the carcinogenicity, neurotoxicity, and dangers to reproductive health associated with chemical contaminants.

**Conclusion:**

To reduce the risks connected to chemical preservatives and food fraud in Africa, stricter regulations and increased public knowledge are required, stakeholders should embrace innovative strategies including tailored education, regional cooperation, and advanced food preservation techniques.

## 1 Introduction

**Figure 1:**
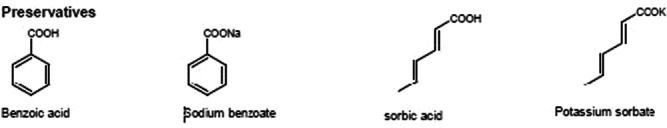
Some common food preservatives. *Source:* Thomas and Adegoke [2].

**Figure 2:**
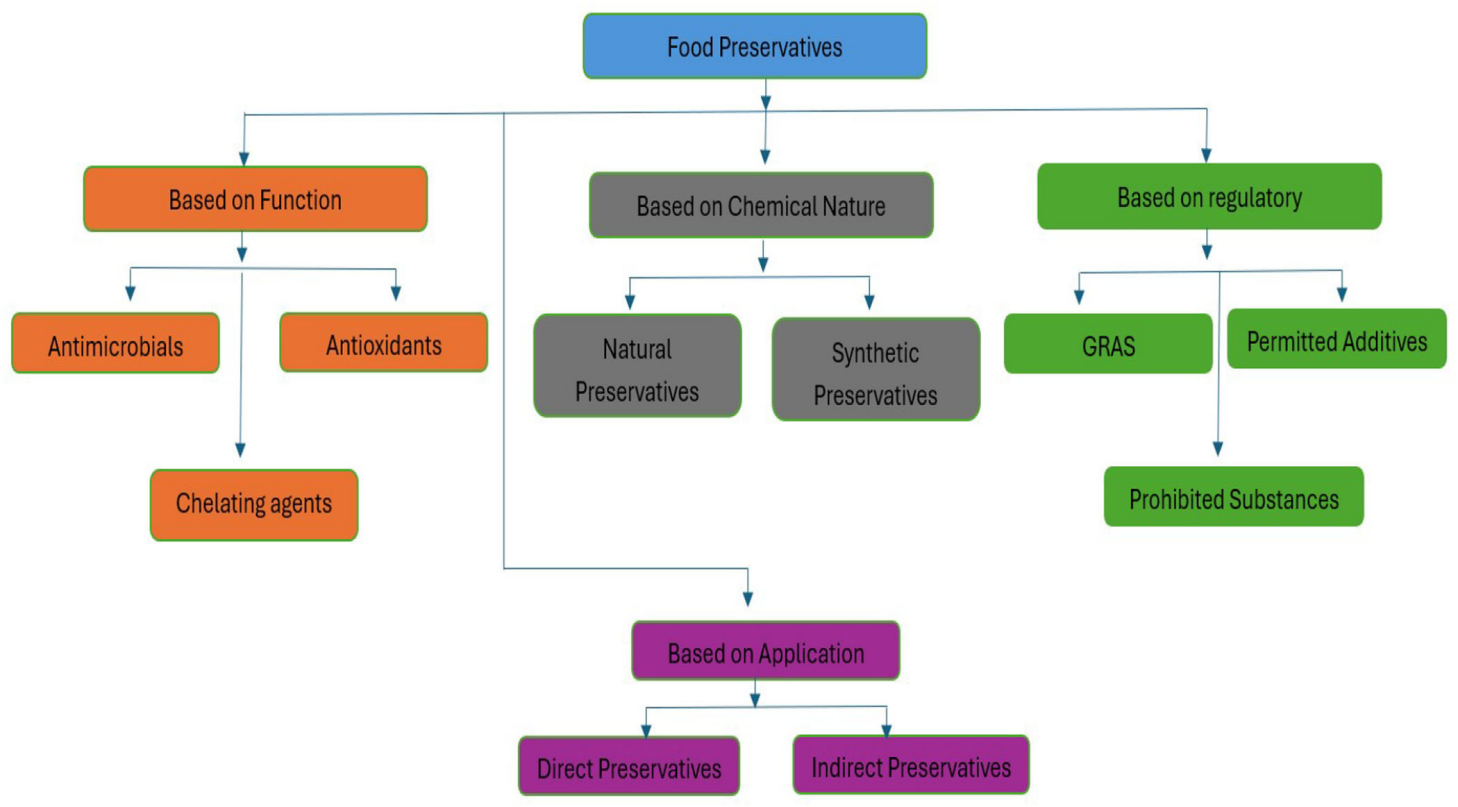
Classification of food preservatives based on origin and function.

Food preservation is crucial for commercial and domestic uses. Majority of the time, food additives are called “preservatives” since they are used to preserve food. When this additive is added, the food's color, texture, flavor, cackle, and other characteristics stay the same for avery long time. Food preservatives are essential for preserving food, but their danger as “slowpoisons” increases the chance of illness or early death [1]. Additives are typically added to foods, beverages, and pharmaceutical items to cover up unappealing colors, obfuscate irritating odors, and improve flavor. Colorants, sweeteners, preservatives, and anticaking agents are examples of food additives [2]. Preservatives are added to these items with the intention of“preventing against” microbial deterioration. Safety considerations are just as significant when using these preservatives. Benzoic acid, benzoate, sorbic acid, sodium and potassium sorbate are some common food preservatives, and so forth. (Figure 1). Food additives may be made synthetically or from minerals, plants, or animals [3]. Figure 2 illustrates the classification of food preservatives. Food preservatives are classified according to their application, chemical composition, regulatory classification, and function. Different regulatory bodies have set specific rules for each category. Preservatives are categorized according to their functions as chelating agents (e.g. EDTA: Ethylenediaminetetraacetic acid), antioxidants (e.g. BHA: Butylated Hydroxyanisole and BHT: Butylated Hydroxytoluene), and antimicrobials (e.g. sorbates, benzoates). In terms of chemical composition, they can be classified into two categories: synthetic (like sorbic acid) and natural (like salt and vinegar). Regulatory classifications, which differ by location and are established by organizations like the Food and Drug Administration (FDA), and European Food Safety Authority (EFSA), represent continuing risk evaluations and scientific studies and comprise generally recognized as safe (GRAS) substances, permitted, and prohibited substances. Direct preservatives, like sodium benzoate in soft drinks and nitrites in cured meats, are added to food to prevent spoilage, while indirect preservatives, such as antioxidants in packaging materials, may migrate into food from packaging or processing.

Preservatives are substances used to inhibit or eliminate the growth of microorganisms in a variety of foods and products [4, 5]. Additionally, preservatives, which can be synthetic or natural, are typically employed in small amounts to stop the growth of food spoilage organisms. Two classes can be distinguished: class I include natural preservatives, and class II contains chemical or synthetic preservatives.

Food preservation is particularly important in Africa for many different reasons. Africa faces enormous food security concerns, with certain regions experiencing postharvest losses of up to 40% due to poor preservation techniques, poor infrastructure, and restricted access to present preservation technologies [6]. These losses intensify food scarcity and increase the need for artificial preservatives to extend the shelf life of food products. Furthermore, Africa's fast‐growing population and urbanization are increasing the demand for processed and preserved goods, which makes the application of chemicals and preservatives more common. However, regulatory systems in many African countries continue to evolve, resulting in gaps in food safety regulations and enforcement. This scenario raises the potential of exposure to harmful chemical toxicants from poorly regulated preservatives, making the preservation of food not only an economic and health concern, but also a public safety issue in Africa.

The food sector has recently developed nontraditional methods to provide high yields at low cost to manufacturers in response to the global need for chemical preservatives brought on by the change in customer needs. High‐energy foods with distinctive flavors and quick preparation have become more popular with consumers [7]. Food industries are therefore caught up in an area of conflict between economy and consumer satisfaction. Food spoilage impacts on economy and business earnings is reduced because, a food with a short shelf life means the food industry may lose production, hence the need for preservatives. However, when used in excessive amounts, these artificial preservatives are harmful to the public's health. In past studies, side effects including headaches, allergies, palpitations, vomiting, and skin rashes were noted [4, 8]. Synthetic preservatives have been shown in other toxicological investigations to be genotoxic and carcinogenic [7].

Chemical toxicants play a crucial role in food preservation, especially in contexts where natural preservation methods are inadequate or too costly. Sodium nitrate (NaNO_3_) and sodium nitrite (NaNO_2_) are chemical toxicants that are frequently used as preservatives for meat products due to their effectiveness in inhibiting bacterial growth. However, these compounds can pose significant health risks. These chemicals are easily converted into nitrous acid under acidic conditions (such as those found in the human stomach) and further decompose to yield nitricoxide (NO) upon reaction with hemoglobin to produce methaemoglobin or nitrosamines, a powerful carcinogen that can cause loss of consciousness [1]. This underscores the challenging balance between the beneficial effects of chemical preservatives in preventing food spoiling and their ability to introduce hazardous substances into the food supply. The risk of exposure to such toxicants is especially high in Africa, where enforcement of regulations is weak and consumer knowledge is poor, making it a crucial issue for food safety on the continent.

Due to man‐made and natural reasons, harmful chemical compounds largely affect the worldwide food sector. As a result, the various steps of food manufacturing are threatened by chemical contamination, endangering food safety.

Developed countries have created novel and strict ways to guide against the contamination of food through food safety management systems [5]. The World Health Organization (WHO) and the Food and Agriculture Organization (FAO) collaborate to establish global food safety standards, encompassing education and safe exposure levels. Their legislative and regulatory efforts aim to ensure the provision of nutritious food to member countries. However, some food industries skimp on adherence to regulations due to strict laws, leading to healthrisks for consumers. Food fraud, deceiving consumers for profit, has historically affected various items such as tea, olive oil, wine, and spices, posing on going challenges. Although majority of fraud occurrences do not represent a risk to the public's health, some have had real or prospective adverse effects [9]. Food fraud can come under the following types namely:

1. Substituting costly components with cheaper alternatives, often by diluting or adding adulterants.
2. Adding minor amounts of fake substances to conceal low‐quality ingredients.
3. Concealing the intentional removal of valuable elements without consumer awareness [8].

The analysis identifies different toxicant problems in different areas: serious health risks arise from the abuse of formalin and sodium benzoate in the preservation of fish and meat in West African countries like Nigeria [10]. Due in large part to a lack of proper regulatory control, Ethephon and Sudan IV are widely used for food coloring and fruit preservation in West Africa, especially Ghana [10]. South Africa is a prime example of the problems Southern Africa suffers with hydrogenated vegetable oils and sodium nitrate in processed meats, which can result in harmful exposure [11]. Health concerns are raised using talcum powder and melamine in food fraud and adulteration in East African [12]. The abuse of sodium nitrite in cured meats, which is made worse by inadequate regulation, is a problem throughout North Africa. Food fraud—intentional adulteration for financial gain—increases public health hazards in all these locations, highlighting the critical need for improved regulatory frameworks and public education on food safety. Given these challenges, this review discusses chemical food preservation in Africa, the potential for fraud, and the associated public health risks. The study focuses on Sub‐Saharan Africa, with particular emphasis on Nigeria, Cameroon, Sudan, Ethiopia, Kenya, Uganda, Ghana, Tanzania, and Congo, exploring the use of chemical toxicants in food preservation and related food fraud activities.

## 2 Methodology

This study focuses on the chemical toxicants used in food preservation in Africa using a comprehensive and systematic methodology including a review of the literature, the data collection, case study analysis, and technological assessment. Each stage is outlined below.

### 2.1 Literature Review

To develop a foundation for understanding the use of different chemical preservatives in food in Africa, an in‐depth literature review was carried out. The review focused on identifying key trends, challenges, and solutions. The following databases were used:

PubMed: For peer‐reviewed articles on food safety and toxicology.

Google Scholar: For a broad range of academic articles and gray literature.

Scopus: For interdisciplinary studies on food fraud detection technologies.

In searching the above databases, keywords such as “food fraud,” “chemical preservatives,” “food safety in Africa,” and “detection technologies” were used. Articles were selected based on actual data, publication in peer‐reviewed journals, and relevance to the topic. The review covered publications published from 2010 to 2023.

### 2.2 Data Collection

Data collection involved gathering quantitative and qualitative data from different sources to gain a holistic view of the subject matter. The primary sources of data included the following:

Peer‐reviewed journals: Providing empirical evidence on chemical preservatives used in Africa, food fraud, and food fraud detection methods.

Government reports: Including national and international food safety guidelines.

Regulatory agency reports focused on food safety regulations, enforcement challenges, and the use of chemical toxicants in food products such as the South African Department of Health and the National Agency for Food and Drug Administration and Control (NAFDAC).

### 2.3 Case Study Selection, Technological Analysis, and Policy Review

To explore the application of modern technologies in detecting food fraud in Africa, case studies were carefully selected based on several criteria. To ensure the relevance of every case study to the local context, the main emphasis was on products that are frequently associated with the food supply in Africa, such as coffee, milk powders, and palm oil. The choice also highlighted the use of cutting‐edge technologies, such as imaging and spectroscopic instruments, which have shown to be successful in detecting food adulteration. To be able to provide an extensive comprehension, case studies were selected from several African nations, which mirror the continent's geographical diversity and the different challenges regarding food safety. A detailed technological analysis was then conducted to evaluate the effectiveness of various detection methods in identifying food fraud. Furthermore, the technological evaluation, a review of food safety regulations and enforcement mechanisms in selected African countries was performed. The analysis focused on countries such as Nigeria, South Africa, and Kenya, each facing distinct challenges in regulating food safety.

## 3 Food Preservation in Africa

Food preservation in Africa is based on traditional methods, which have evolved in the African communities and have been transferred from one generation to another [7]. These indigenous behaviors stem from a cultural relationship with certain conditions of the environment, and they are founded on traditional communities' close awareness of their surroundings [13]. Food is primarily stored at the household level in traditional architectures in African countries [14].

Farmers adopt food preservation techniques such as drying and the use of moisture‐proof and sufficiently aired storage structures to avoid losses after harvesting [15]. Clay rhombus, cribs, earthen pots or baskets, granaries, platforms, thatched rhombus, and domestic or interior storage such as earthen pots, gourds, metal containers, and plastic containers are examples of these structures. Bags constructed of hessian, jute, plant fiber, or polyethylene are examples of other storage systems [14]. This section is dedicated to briefly re‐examining the different African food preservation practices adopted by farmers.

## 4 Drying

Drying is among the oldest traditional ways of food preservation; it is important for food preservation because it reduces the amount of water in food items to a tolerable amount and extends the shelf life of the food [16, 17]. Drying is also a step in the food processing process; for example, foods must have been dried before being processed into flour [7]. Sun drying is accomplished by spreading under the sun, fresh produce on bareground, rooftops, or roadsides; it is a low‐cost method of food preservation due to its adaptability to a wide range of meals [18]. Examples of sun‐dried foods include peeled yam, peeled cassava, maize, vegetables, and so on. Meat and fish are smoked to improve flavor while also enhancing shelf life [7, 19]. Tomatoes are preserved by sun drying in Nigeria. Dried tomatoes can be stored for up to 12 months. In Sudan, meats are sliced into long pieces, salted, sprinkled with powdered coriander, and sun‐dried to produce “shermout” [20].

## 5 Storage Bags

The use of sacks in the preservation of grains is common in Africa. Sacks made from cotton, native grass, sisal, woven jute, and other materials based on what is available in the vicinity. Farmers still use sisal or jute bags. Polyethylene storage bags are also used in providing a hermetic storage environment for food products. To maintain water resistance and complete airtight storage conditions, a polyethylene bag is inserted in storage bags for an additional protective layer to make multilayer polyethylene storage bags [21, 22].

## 6 Curing and Storage With Table Salt

Table salt is used by rural farmers to store*Phaseolus vulgaris*and *Cajanus cajan*. About 200 g of salt is properly mixed with 1 kg of *P. vulgaris* and *C. cajan*. The essential concept of this approach is that insect infestation and population are reduced because of abrasive effect of salt, which restricts insect mobility in the storage container [23].

The main idea behind curing certain food products like fish, meat, and vegetables is to lower moisture content through the process of osmosis. When the moisture content of any food is kept to a minimum, the chances of microbial contamination and subsequent growth are significantly lowered [24]. Curing can also be used to flavor food, this is done by combining sugar, salt, nitrates, and nitrites in amounts sufficient to dewater the food. Higher salt content in curing kills bacteria by dehydrating them. Salt also can reduce oxidative degradation, resulting in slower fat oxidation and, as a result, reduced rancidity [25].

## 7 Boiling

Boiling is commonly used to kill microorganisms in food; the food is then cooled before consumption [26]. It is also common to boil milk before drinking it to destroy any bacteria that may exist [27]. Boiling meat products changes its chemical and physical properties, improving the quality and shelf life [28]. It is done to destroy or reduce the contaminating microflora that accumulates during the slaughtering processes. Boiling is also used before eating to improve texture, flavor, and color [29, 30]. Since man discovered the use of heat in processing and preserving food, boiling has been widely employed as an effective method of preservation.

## 8 Fermentation

Fermentative microorganisms used in fermentation defend the food from other microbial pathogens by creating an acid or alcohol that is harmful to them. Throughout fermentation, parameters such as oxygen level, salt, temperature, and other parameters are maintained, enabling the fermentative microbe in the production of a food product fit for consumption [25].

Meat fermentation is employed to create favorable indigenous flora, such as color, flavor, and taste [31]. Backslopping was a frequent procedure in the fermentation of meat, which included adding small amounts of previously fermented meat with strong sensory characteristics. This method produces fermented meat of varying quality [30]. In terms of quality, traditionally fermented meat is still considered superior to the industrial fermented meat. In meat fermentation, salty mince and fat are typically pressed together in casings to generate anaerobic conditions. Meat fermentation is mainly done using hog, beef, and mutton [32].

Other food preservation techniques used in Africa include storage with diatomitzed earth, open air/aerial storage, storage in gourds, grain storage in Palmyra leaf (*Broassuslabellifer L*.) bin, storage of grains in mud silos and underground pit, and so forth [14].

## 9 Chemical Toxicants Used in Food Preservation

The use and addition of different chemicals as food preservatives prevents wastage by protecting food from microbial spoilage and thus, maintaining freshness by preventing changes in quality. However, when used excessively, preservatives may raise some adverse effects relating to human health. Table 1 is used to provide an overview of chemical toxicants in food preservation and fraud.

**Table 1:** Overview of chemical toxicants in food preservation and fraud.

Toxicant class	Common uses	Health risks	Examples of misuse in Africa	Notes/References
**Preservatives**	Prevent spoilage, extend shelf life	Allergies, carcinogenicity, genotoxicity	Sodium nitrate/nitrite in meat (converts to nitrosamines)	Misused in high quantities, especially in meats [11]
**Colorants**	Enhance appearance of food	Carcinogenicity (e.g., Sudan IV), allergic reactions	Sudan IV in palm oil, fake food colorants	Often used to mislead consumers about food quality [10]
**Antimicrobials**	Inhibit microbial growth	Possible development of antibiotic resistance, toxicity	Formalin in fish and milk, excessive sodium benzoate in bread	Formalin used inappropriately in various products [10]
**Sweeteners**	Improve taste	Metabolic issues, potential carcinogenic effects	Added to honey to increase sweetness	Often used to deceive consumers about product quality [33]
**Flavor enhancers**	Enhance or modify taste	Neurotoxic effects (e.g., MSG), gastrointestinal issues	Excessive use of MSG in local foods	Used to boost flavor without proper labeling [34]
**Ripening agents**	Accelerate ripening of fruits and vegetables	Health effects vary depending on agent used	Ethrel (ethephon) for fruit ripening in various countries	Used to manipulate ripeness, affecting quality [10, 35]
**Solvents**	Used in extraction processes or as preservatives	Toxicity to liver, kidney damage	Use of industrial chemicals like melamine in milk	Melamine added to increase apparent protein content [12]
**Curing agents**	Preserve and flavor meats and fish	Risk of carcinogenic compounds, health issues	Excessive salt and nitrate in cured meats	Used to extend shelf life but can lead to health risks [36]

Developing countries are faced with the challenge of inadequate knowledge of the exact amount of chemicals to add, leading to adverse health conditions. Direct antimicrobial preservatives are substances that are added to food for the goal of food preservation due to their chemical properties and established antibacterial activities [37]. Benzoates, nitrate, nitrite, propionates, sorbates, and sulfites are examples of synthetic chemical agents [38]. Indirect antimicrobial preservatives, on the other hand, are compounds added to food for purposes other than their antibacterial capabilities. These include flavor and color stabilizers with supplementary traces of microbial growth inhibitory effect.

Some chemical preservatives can be toxic, for example, while benzoic acid is normally mild intoxicity, large quantities can cause acid/base imbalances and disrupt intermediary metabolism [39]. Research has revealed that in the presence of erythorbic acid or ascorbic, benzoates can undergo decarboxylation, producing the carcinogenic chemical “benzene” [40]. Nonetheless, the application of benzoic acid and its sodium and potassium salts in food preservation is widely regarded as safe, with no evidence of genotoxicity or carcinogenicity [39]. Experimental discoveries have demonstrated a link between nitrate and nitrite consumption and certain cancer types [41]. It is possible that the carcinogenic potential of this family of food preservatives is due to the degradation of nitrites and nitrates to nitrosamine [42]. Sulfites have been linked to a variety of unpleasant clinical effects in sensitive people, including abdominal pain, dermatitis, diarrhea, flushing, hypotension, and urticaria, and potentially fatal anaphylactic and asthmatic reactions.

Scientists and food inspectors have discovered that toxic chemicals are used by food vendors across Sub‐Saharan Africa to enhance the appearance of meat, fruits, and vegetables. While some fishermen pour gamalin (a toxic insecticide) into water in countries like Cameroon and Nigeria [43] leading to the death of aquatic life‐forms present and the gathering and subsequent sales of the fish, hunters apply formalin (a chemical used for embalming and preserving bodies in morgues) on the bodies of their killed animals to keep them from decomposing until they return to their villages [35]. In Ethiopia, it was also reported that the farmers added formalin in what is known as “milk medicine,” which is added to the milk for preservation before selling to factories. Researchers report that formalin and some chemicals used for extending the shelf life of fruits can cause dizziness, weakness, ulcers, heart disease, skin disease, lung failure, cancer, and kidney failure [35]. There is also the addition of phytosanitary agents to quicken the ripening of pineapple, banana, and plantain after harvest in some African countries such as Cameroon and Nigeria [43]. Agents such as ethephonal so referred to as Ethrel or the plant hormones gibberellins are used indiscriminately to achieve this purpose [35]. In a study [44], sodium benzoate, benzoic acid and other chemically defined preservatives were used at a dosage recommended by the Food and Drug Administration, USA in the extension of the shelf‐life of a locally produced flatbread, Injera, which is a staple food in Ethiopia, from 3 to about 12 days. Unfortunately, illegal, and unprofessional locals add large doses of these chemicals into Injera by trial and error at the expense of human health.

### 9.1 Analysis of Chemical Toxicants Induced Effects in Africa

The effects of chemical toxicants on food safety in Africa is a critical public health issue that has been researched to comprehend the magnitude and ramifications of these impacts. This section presents some statistical summary of the documented effects of chemical toxicants on the African population, based on current research and available data.

Due to their widespread use in agriculture, pesticide residues are a major concern in Africa. Research by the WHO and the FAO found that in about 10% of food samples tested across several African countries, pesticide levels were found to be above the maximum permissible limits [45]. In a 2019 study of fresh food in Kenya, 22% of samples tested positive for pesticide residues above allowable limits; the most prevalent pollutants were organophosphates [46]. According to a study by Anaduakaet al. [47], there are increasing cases of leukemia and lymphomas, among other malignancies, in agricultural populations who are exposed to pesticide residues. Chronic pesticide exposure has also been connected to reproductive health problems and neuro developmental impairments. According to a study conducted in Nigeria, children who grow up in agricultural areas are more likely to experience developmental delays than children who grow up in nonagricultural areas [48].

Contaminants and food additives increase the risk to one's health. Food samples from South Africa's major market places included food additives that were not listed on product labels [49]. Preservatives and colorants are common examples of these unlisted additives, and consuming high amounts of them may be harmful to the body's health. For instance, Ghanaian chili powder samples were reported to contain Sudan I, a synthetic colon that is prohibited in many nations [50]. Sudan dyes has been connected to cancer‐causing effects, raising worries about regulations and calls for more stringent monitoring. Foodborne illnesses resulting from chemical toxicants are likewise a major concern [51].

### 9.2 Food Safety Regulations and Standards in Various African Countries

The legislation pertaining to food safety in African nations reveal significant variation, which can be attributed to varying degrees of development, resources, and regulatory proficiency. When compared to other African countries, the regulatory system in South Africa is quite strong. The cornerstone of South Africa's food safety laws is the Foodstuffs, Cosmetics, and Disinfectants Act (Act No. 54 of 1972), which includes extensive rules on food standards, labeling, and safety [52]. Regulations that comply with international standards, such as those issued by the Codex Alimentarius Commission, are an addition to this Act. High‐risk food industries are required to use the Hazard Analysis and Critical Control Points (HACCP) system, which helps to guarantee that food safety is maintained across the food production process.

However, the regulatory environment for food safety in Nigeria is more fragmented. Food safety in Nigeria is mostly supervised by the National Agency for Food and Drug Administration and Control (NAFDAC) [53], however due to overlaps with other organizations, such as the Standards Organization of Nigeria (SON), NAFDAC's efficacy is frequently compromised. Inconsistent enforcement and gaps in regulation may result from this fragmentation. Nigeria's food safety regulations intend to conform to global standards; yet obstacles such insufficient infrastructure and restricted resources sometimes impede its execution [54].

Kenya and Uganda have taken significant steps in the East African region to align their food safety laws with the regional guidelines established by the East African Community (EAC). Kenya's Public Health Act and the Food, Drugs, and Chemical Substances Act provide the framework for food safety in the country; the Kenya Bureau of Standards (KEBS) is primarily responsible for establishing and implementing food safety regulations [55]. The Uganda National Bureau of Standards (UNBS) is in charge of regulating food safety in Uganda, which is governed by the Food and Drugs Act [56]. Even with these frameworks, enforcement has remained inconsistent, especially in rural areas and informal markets. This underscores a larger problem with resource allocation and regulatory capacity. Many African countries have outdated or nonexistent regulatory systems many African countries who have recently updated their legislation have recognized the need, as a minimum, to establish a formal system of collaboration [57]. The absence of current food safety rules and enforcement procedures adds to widespread food safety concerns. The WHO [58] emphasizes the serious weaknesses in these nations, where a lack of infrastructure, along with political instability, impedes efforts to develop and enforce adequate food safety regulations.

## 10 Food Fraud in Africa

Food fraud is a deliberate act to adulterate or falsify food to maximize profit. It could be in several forms, including changing the color, taste, texture, addition of illegal additives, concealment, misrepresentation of ingredients, dilution, counterfeit, unapproved enhancement, measurement fraud, food substitution, fake branding, extended use‐dates on cans/packed foods and mislabelling. It could be because of the oversight shortfall or even lack of it, little or lack of regulations and the extensive informal markets within this region [59]. Additionally, pressure onproducers to meet demands, increased competition from cheaper products, inflation, and food shortage [60, 61]. Africa's food imports hit about 30 billion dollars annually [62], and more than 50% of all goods, including foods, drugs, and other materials imported into Tanzania are reportedly fake [60]. So African communities could be flooded with fraudulent foodstuffs. Food fraud not only has economic implications but also endanger the lives of consumers.

There are several foods malpractices in Africa including the use of formalin (embalming agent) to treat fish and meat to falsify their freshness in Uganda, Cameroon and Congo. Furthermore, the acceleration of fruits ripening with Ethrel across the continent, and the use of sodium benzoate to improve the shelf life of bread in Ethiopia and the addition of Sudan IV (a fat–soluble dye–carcinogen) as a food coloring agent in palm oil in Ghana [63]. Butter adulteration in Ethiopia [64], with hydrogenated vegetable oils, maize dough, wheat dough, Irish potato puree, melted tallow, banana pulp, buttermilk, and even water. More so, there are reports of the addition of sweeteners (sucrose and sugar) in honey to sweeten the product [65]. Adulteration of powdered milk products exported and sold in East African markets with melamine [33], an industrial chemical used as a fire retardant and as a plastic stabilizer. Melamine is rich in nitrogen, and its addition to milk can make it appear to contain high levels of protein because of the high nitrogen content of melamine [33]. Chia oil adulteration with food oils like sunflower, corn, and rapeseed oils [12]. Mostly fish and fish products, meat and meat products, milk and milk products, palm oil, packed foods, canned foods, powdered products (cereals and legumes), wine, spices, and honey are implicated as Africa's most adulterated food products.

The rising consumption of processed meat products like sausages has fueled fraud in the meat industry, allowing for substitution of high‐quality meat with lower‐quality alternatives [66]. Food mislabelling, substitution, and concealing could cause consumption of food prohibited in some regions and religions, food allergens, or even death. Globally, about 57% of people out of which 32% are children are estimated to develop health challenges from consuming adulterated food [65]. However, fortification of food for the sole purpose of improving the nutritional contents of food and meeting the nutritional needs [62] could be acceptable when the contents of the said prepacked food are listed for consumers to make an informed decision as to whether to consume the said product. Although food fortifications present some nutritional benefits, especially the addition of micronutrients, they could pose a risk of exposure to food allergens, and food safety concerns, especially when consumed without knowledge of the food content.

## 11 The Link Between the Use of Chemical Toxicants in Food Preservation and Food Fraud

Foods are made up of chemical compounds, which form our nutrients (e.g., proteins), fibers (e.g., cellulose), micronutrients (e.g., vitamins), and are essentials for good health. Yet, either deliberately or unintentionally, some chemicals are introduced in to our foods which turnout to be harmful when consumed [63, 67]. The desire to prolong food shelf‐life drove the development of food preservation methods. This advancement in food preservation and the complexities of modern supply chain resulted in another challenge of protecting the consumers from chemical substances, or chemical toxicants used in food preservation [68, 69].

The soil, environment, water, air, packaging material, and microbial contamination are amongst the various sources chemical toxicants can get into our foods. Chemical toxicants in food can either be organic or inorganic [70, 71, 72]. Not all chemicals employed in food preservation represent a risk to consumers as chemical toxicants [73]. And food contamination with chemical toxicants can be inadvertent without the intent of causing harm [63]. However, there exists the likelihood that certain people can choose to take advantage of gaps in food supply chains for economic gains, without considering the public health impact of their actions [74, 75]. The intentional act of adulterating food for economic gain, whether or not such act leads to food safety threats and public health food risk is regarded as food fraud [75]. Intentional introduction of chemical substances that may or may not be GRAS owing to their nature and/or quantity in food production can serve as a major source of chemical toxicants to the consumers. And foodborne diseases caused by chemical contamination represent some of the most prevalent sources of food contamination [74].

While most fraud cases do not pose a public health risk, over the years, there has been cases of fraudulent adulteration of food and feeds [76] with chemical substances along the supply chain that has led to serious health crisis, and fatality in some cases. Many people believe that the true estimate of food fraud impact cannot be determined because of the intentionality of the perpetrators in evading detection, however, few high‐profile cases have made the news round [77]. Infant milk formula has been adulterated with melamine because of its property to increase the apparent level of protein content, resulting in the death of six peoples with thousands falling ill. The unlawful use of the carcinogenic red dye Sudan red 1 in chicken products, and the use of clenbuterol hydrochloride in animal feed resulted in many foodborne illnesses [78, 79]. Toxic chemicals such as sulfur dioxide, Sudan red, fluorescent bleacheer, di(2‐ethylhexyl) phthalate(DEHP), alum, monosodium glutamate (MGM or MSG) and talcum powder are added to food stochange either the color, appearance, or texture. MGM is an adulterant that is added to foods to improve the flavor and give the umami taste to the food. However, MGM contains l‐glutamate and has been shown to have neuroexcitatory action. DEPH and other related phthalates are associated with cancerous and reproductive health risk concerns [80]. The chances of adulterating food products with toxic chemicals for economic gains to the detriment of human and animal health represent a major food industry issue and a public health challenge to be resolved [71].

## 12 Understanding the Toxicological Impact of Commonly Used Chemical Toxicants in Foods

Studies have discovered a link between prenatal exposure to polychlorinated biphenyl (PCB) and dioxin and aspects of cognition important to language in early infancy. Caspersen et al. [34]. discovered a link between maternal exposure to PCB‐153 or dl‐compounds and deficient grammar indicating as light loss in language skills in both girls and boys. Prenatal PCB and dioxin PCB exposure may be detrimental to a developing fetus and raise the risk of impaired or delayed neurodevelopment. Girls born to mothers who consumed more than the tolerable weekly dl‐compound consumption or a PCB153 intake above the 97.5percentile during pregnancy may have an increase in the risk of language impairment at the age of three.

Owing to the use dichloro‐diphenyl‐trichloroethane (DDT) in the reduction of mosquitoes, the contamination of the marine habitat with DDT and the subsequent intake of DDT contaminated marine products, a study was done to estimate the human health risk associated with the consumption of DDT‐contaminated marine fish from Maputo Bay. Thompson et al. [81] reported a median of 3.8 ng/gww (maximum 280.9 ng/gww) for ∑DDTs. At the 75th percentile concentration, the total hazard ratio was 1.5, and at the 95th percentile, it was 28.2. These calculations suggest that DDT contamination increases the risk of cancer.

People who eat a lot of fish and shell fish may be exposed to a significant amount of methylmercury [82]. Nearly 85% of methylmercury consumed is absorbed in the gastrointestinal system, 5% in the bloodstream, and about 10% in the brain. Most populations are exposed to mercury due to the ingestion of fish and other seafood contaminated with mercury [83]. For decades, mercury's harmful effects were mostly connected with the central nervous system. An increasing body of research, however, reports that exposure to methylmercury can raise the risk of deleterious cardiovascular effects in exposed groups [84, 85, 86]. Mercury and methylmercury cause mitochondrial dysfunction, reduce ATP production, deplete glutathione, and enhance the peroxidation of DNA, protein, and phospholipid [87].

Polycyclic aromatic hydrocarbons (PAHs) have been linked to health hazards, particularly cancer [88]. Consumption of contaminated food, which could happened during processing and preparation, is one form of contamination by these compounds (i.e., environmental pollution). Unlike changes produced by microbial features and lipid oxidation, people cannot detect the Presence of PAH in food products, limiting their capacity to reject these foods. PAH absorption is aided by their increased fat solubility, and their lipophilic features help them in adhering to the cell membrane. This binding alters the structure of the cell, disrupting normal cell functioning [89]. Among PAHs, benzo[a]pyrene is most easily soluble in lipids. PAHs may bind to lipid distribution molecules like the chylomicrons and other lipoproteins, allowing it to enter several systems involved in lipid absorption and dissemination and, as a result, promote its bioaccumulationin organs and tissues like the liver and small intestine [90].

Importantly, prolonged human exposure to PAHs can explain why some dieases, such as intestinal disorders in non‐smokers and lung cancer in smokers, are becoming more common [91, 92]. Foods high in lipids (e.g., fish, beef, and chicken) are effective delivery routes for these molecules, facilitating absorption via the gastrointestinal tract [93, 94].

PAHs can produce chromosomal mutations, disrupt junction and fusion processes, and potentially trigger chromosomal breaks. As a result, making the genetic material present in these cells unstable. If mechanisms of DNA repair are not activated, the genotoxic substance will permanently damage the cell's DNA at the start of the transcription phase, resulting in an irreversible mutation and a preneoplastic cell [95].

## 13 Current Approach to Managing Chemical Toxicants in Africa's Food System

To manage chemical toxicants in Africa's food system, it is pertinent to implement food safety standards and policies to guide food production and distribution [96]. African food policymakers must set high regulatory food safety standard, enforce transparent labeling of contents, adhere to additive and preservative regulations, and ensure ecofriendly packaging while preventing chemical contamination in all stages of production and distribution [5]. Therefore, to monitor these processes in food production and distribution, there is a need to employ rapid, and efficient analytical systems to identify food risks on time at any stage of food production and distribution.

Food contents can be authenticated before consumption and possible contaminating chemical toxicants could be detected using a variety of analytical techniques. A screening technique that employs a direct analysis of sample to get her with a time‐of‐flight (TOF) mass spectrometer could identify Sudan dyes I to IV in tomato sauce, palm oil, and chilli powder [97]. This screening technique was reportedly sensitive with a limit of identification in compliance with the limits identified by the European Union for analytical methods (0.5–1 mg/kg). Others including surface‐enhanced Raman spectroscopy and chemometrics were used to authenticate edible palm oil and detect adulteration with Sudan dyes [98]. Portable near‐infrared spectroscopy (NIRS) and chemometrics were used to identify palm oil which has been adulterated with Sudan dyes and differentiated them from the authentic ones [99]. With NIRS and chemometrics, it was possible to classify and authenticate rice samples [100], analyse, differentiate, and authenticate conventional and organic cocoa beans produced in Ghana [101], differentiate species and muscle type of South African games including treatments (fresh or previously frozen) [102]. More so, NIRS, chemometrics, microbiological and packaging parameters were used to authenticate fat‐filled milk powders (FMP), a low‐cost milk alternative exported to underdeveloped countries for the presence of urea and melamine, including confirming the freshness and stability of the product [103]. Nuclear magnetic resonance (NMR) spectroscopy was used to detect possible methanol mixtures in brand spirits and differentiated counterfeited products from authentic ones [104]. NMR was reportedly used in the metabolic characterization of coffee samples and promising in the determination of quality and detection of fraud in food matrices [105]. Inductively coupled plasma (ICP) and x‐ray fluorescence (XRF) spectrometry was used to discriminate Ethiopian coffee based on its geographical origins and its promise in the detection of fraud in the food sample [106]. Using an ultrahigh‐performance liquid chromatography‐quadrupole time of flight tandem spectrometry (UHPLC‐QTOF‐MS) targeting a set of phenolic compounds, it was possible to differentiate between Greek PDO kalamata table olives and otherkalamata table olives from Egypt and Chile [107]. Using spectroscopic analysis, it was possible to detect adulteration in chia oil with sensitivities, accuracies, and specificities of over 90% [12]. In addition, these spectral and imaging technologies including vibrational spectroscopy (NIRS, Raman spectroscopy), hyperspectral imaging, color imaging, x‐ray imaging and computed tomography as discussed elsewhere [66], provides a rapid nondestructive method for evaluating meat and meat products for fraud detection.

The food industry requires surveillance and rigorous toxicological research using reliable tools to detect chemical toxicity in food, enhancing quality and safety for consumers. Analytical testing tools, like nondestructive methods, offer real time monitoring and accurate evaluation for adulteration and food fingerprinting. This highlights the need for African food policymakers and regulatory agencies to invest in these modern tools to monitor and promptly address food malpractices in the region's food supply chain.

## 14 Guiding Against the Use of Chemical Toxicants in Food Preservation

In Sub‐Saharan Africa, the issue of food safety and food‐borne toxicants is aggravated by public misunderstanding, an uncoordinated approach to food management, a lack of technical knowledge and inadequately equipped facilities in some circumstances, and poor enforcement of laws and regulatory restrictions. National food control systems are developed to satisfy specific country's needs and goals [43]. They may differ across countries, but to be effective, they must include key components such as food regulations and legislation, policy and institutional frameworks, food inspection and surveillance, food laboratory services, participation of relevant stakeholders, and dissemination of information to them. To improve food safety, the Nigerian government has set up certain organizations. Such bodies include the Federal Ministry of Health, the Standard Organization of Nigeria, The National Agency for Food and Drug Administration and Control (NAFDAC) to name a few [43].

Toxic concerns in consumer products ranging from food, herbal medicine, drugs, and cosmetics have been widely recorded throughout Africa. Less than nine African countries out of 54 have a legally recognized toxicovigilance system [108]. Morocco, Algeria, Benin, Ghana, Morocco, Senegal, and the Republic of South Africa are examples of such countries. While Kenya and Zimbabwe are working toward a toxicovigilance system through established Poison Information Centres, Cameroon with no organized activity in the field yet has set up practices for monitoring specific chemicals of concern, and a response plan in preparedness to an outbreak.

The growing number of reports involving the misuse of food additives and chemicals prompted Kenya's Ministry of Health and Agriculture to begin plans for a National Food Safety Authority. In addition to the obligation for authorities to conduct frequent food safety inspections, experts agree on the importance of increased knowledge among producers of fruits and vegetables, fish, and meat as well as consumers [109].

### 14.1 Enforcement of Regulations and Their Effectiveness

The enforcement mechanisms in place have a major impact on how effective food safety standards are in Africa. Owing to the Foodstuffs, Cosmetics, and Disinfectants Act, which provided a comprehensive framework, food safety standards are enforced relatively well in South Africa. Environmental Health Practitioners (EHPs) at the National Department of Health (Port health) and Metropolitan and District municipalities (Municipal Health Services) are the primary stakeholder responsible for ensuring compliance with food safety laws in SA. Achieving thorough monitoring is challenging due to the shortage of inspectors and laboratories in addition to the magnitude of the food industry and the widespread presence of unofficial food vendors [52]. For example, the study by Yahaya et al. [110] underscores the challenges faced in ensuring the safety of fruits and vegetables. This study employing structured questionnaires were used to collect demographic information from 50 vegetable and fruit vendors within selected markets in Lagos in examining chemical preservatives vegetable and fruit merchants in Lagos markets used. The study found that 44% of them used dangerous chemicals like DDT, sniper, and gamalin‐20, with the extensive usage of these preservatives found to pose a serious health risk.

Enforcing food safety rules in Nigeria is fraught with difficulties. The work of regulatory organizations like NAFDAC is frequently hampered by inadequate budget, inadequate facilities, and corruption. According to Ukwueze [111], the existence of several regulatory bodies with overlapping powers may result in inefficiencies and uneven enforcement. These enforcement mechanism flaws contribute to the prevalence of food safety issues such adulteration and the selling of subpar products. Kenya also faces difficulties with enforcement, especially in informal and rural marketplaces. Though they actively monitor food safety, the KEBS and other regulatory authorities have limited reach outside of urban regions. The efficiency of regulatory measures is compromised by corruption and the influence of influential industry stakeholders [112]. Enforcement organizations may confront challenges related to logistical problems and limited resources.

Enforcement is essentially non‐existent in some African nations, for example Ohene‐Darko et al. [113] identified the need for stronger institutional capacity building, effective collaboration among stakeholders, sufficient regulator food service operator contacts towards improving food safety in Ghana. Ineffective food safety procedures are the result of severe resource restrictions combined with a lack of political will. Food safety problems are made worse by the lack of routine laboratory testing, inspections, and public awareness campaigns, which frequently results in food product contamination [114]. These difficulties draw attention to the necessity of raising political commitment and funding regulatory infrastructure to enhance food safety enforcement throughout the continent.

## 15 Conclusion and Recommendation

Chemical food preservatives have garnered extensive acceptance due to their accrued benefits over time; however, their deleterious effects in the food sector remain predominant. It is not yet absolute to claim that chemical toxicants used in food preservation are intended toward fraud; however, food fraud is more likely than not, in the African context. Ignorance on the part of rural locals and economic benefits on the part of the industrial sector contribute to the rather unintended negative health impacts. Therefore, we conclude that chemical toxicants used in preservation are partly due to ignorance and partly a case of food fraud. The need for global awareness of the integrity of food consumed in Africa cannot be overemphasized. Although there are few individual reports on food fraud in Africa, unfortunately, there is little or no scientific evidence to support them. Moreover, knowing the problem is not enough, all need to be part ofthe solution. This calls for all stakeholders, including consumers to be part of the solution. Therefore, African policymakers and food regulatory agencies need to devise innovative approaches to tackling the associated health impacts of food fraud. A number of innovative strategies should be adopted by stakeholders to enhance the effectiveness of food safety regulations. These include developing tailored educational campaigns that make use of materials and languages unique to the area to improve comprehension between industry workers and rural communities; putting in place innovative, portable detection technologies for identifying toxic chemical residues throughout the food supply chain; and enhancing regional cooperation among African nations to create a single regulatory framework and exchange best practices. Furthermore, promoting research into natural preservatives and traditional methods, improving transparency and traceability in the food supply chain, and rewarding ethical industrial practices will help to match economic gains with public health goals. Moreover, the adoption of innovative food preservation and storage technologies has the potential to reduce reliance on chemical preservatives while also addressing food fraud. These methods include the use of nanomaterials in improving the effectiveness of natural antimicrobials [115], developing eco‐friendly biopolymer‐based packaging [116], the use of antimicrobial peptides as natural preservatives [117], and the application of nanotechnology in the production of bioactive metabolites [118]. Furthermore, the use of green chemistry in producing antimicrobials has demonstrated promise as safe preservatives, toxic chemical adsorbents, and biocompatible solutions [119].

## Author Contribution

Nnabueze Darlington Nnaji: conceptualization, first draft preparation, editing, and supervision. Helen Onyeaka: conceptualization, first draft preparation, editing, and supervision. Kingsley Tochukwu Ughamba: firstdraft writing and final correction. Chukwuebuka Maxwell Ononugbo: writing and proofreading. Chinasa Valerie Olovo: writing and proofreading. Ifeanyi Michael: writing and proofreading. All authors read and approved the final article.

## Consent

All authors consent to the publication of the article.

## Conflicts of Interest

The authors declare no conflicts of interest.

## Data Availability

The authors have nothing to report.

## References

[1] D. Mandal , “Food Preservative Chemistry: Effects and Side Effects,” Journal of theIndian ChemicalSociety 96, no. 12 (2019): 1519–1528.

[2] O. E. Thomas and O. A. Adegoke , “Toxicity of Food Colours and Additives: A review,” African Journal of Pharmacy and Pharmacology 9, no. 36 (2015): 900–914.

[3] C. G. Awuchi , H. Twinomuhwezi , V. S. Igwe , and I. O. Amagwula , “Food Additives and Food Preservatives for Domestic and Industrial Food Applications,” Journal of Animal Health 2, no. 1 (2020): 1–16.

[4] D. Jayant and P. M. Halami , Current Developments in Biotechnology and Bioengineering. Industrial Perspective of Food Preservatives From Microbial Origin. (Amsterdam, The Netherlands: Elsevier, 2020), 243–261.

[5] K. Lebelo , N. Malebo , M. J. Mochane , and M. Masinde , “Chemical Contamination Pathways and the Food Safety Implications Along the Various Stages of Food Production: A Review,” International Journal of Environmental Research and Public Health 18 (2021): 5975. 34071295 10.3390/ijerph18115795 PMC8199310

[6] G. O. L. Bruna , A. C. C. Thais , and A. C. C. Lígia , “Food Additives and Their Health Effects: A Review on Preservative Sodium Benzoate,” African Journal of Biotechnology 17, no. 10 (2018): 306–310.

[7] J. Okoye and K. Oni , “Promotion of Indigenous Food Preservation and Processing Knowledge and the Challenge of Food Security in Africa,” Journal of food security 5, no. 3 (2017): 75–87.

[8] A. A. Kamal and S. A. S. Fawzia , “Toxicological and Safety Assessment of Tartrazineasa Synthetic Food Additive on Health Biomarkers: A Review,” African Journal of Biotechnology 17, no. 6 (2018): 139–149.

[9] R. Johnson , “Food Fraud and “Economically Motivated Adulteration” of Food and Food Ingredients,” Congregational Research Service (2014): 1–45.

[10] H. Onyeaka , M. S. Kalane , A. T. Guta , and P. Tamasiga , “Food Fraud Amid Covid‐ 19 in Sub‐Saharan Africa,” Public Health in Practice (Oxford, England) 3 (2022): 100234. 36101763 10.1016/j.puhip.2022.100234 PMC9461351

[11] E. Mkhwebane , J. Bekker , and N. Mokgalaka‐Fleischmann , “Sodium Reduction in Processed Foods, Including Processed Meats, in Africa: A Systemic Review,” African Journal of Food, Agriculture, Nutrition and Development 23, no. 3 (2023): 22730–22750.

[12] D. Schoder , “Melamine Milk Powder and Infant Formula Sold in East Africa,” Journal of Food Protection 73, no. 9 (2010): 1709–1714. 20828481 10.4315/0362-028x-73.9.1709

[13] C. Perez , E. M. Jones , P. Kristjanson , et al., “How Resilient Are Farming Households and Communities to a Changing Climate in Africa? A Gender‐Based Perspective,” Global Environmental Change 34 (2015): 95–107.

[14] A. J. Mobolade , N. Bunindro , D. Sahoo , and Y. Rajashekar , “Traditional Methods of Food Grains Preservation and Storage in Nigeria and India,” Annals of Agricultural Sciences 64, no. 2 (2019): 196–205.

[15] T. M. Nduku , H. De Groote , and J. M. Nzuma (2013). Comparative Analysis of Maize Storage Structures in Kenya (No. 309‐2016‐5259).

[16] N. Ahmed , J. Singh , H. Chauhan , P. G. A. Anjum , and H. Kour , “Different Drying Methods: Their Applications and Recent Advances,” International Journal of food nutritionandsafety 4, no. 1 (2013): 34–42.

[17] K. S. Jayaraman and D. D. Gupta , Handbook of Industrial Drying (Boca Raton, FL: CRC Press, 2020), 643–690. Drying of Fruits and Vegetables.

[18] M. U. Joardder and M. H. Masud , “Food Preservation Techniques in Developing Countries.” Food Preservation in Developing Countries: Challenges and Solutions (Cham: Springer, 2019a), 67–125.

[19] M. K. Walingo , “Indigenous Food Processing Methods That Improve Nutrient Bioavailability Inplant‐based Diets of the Kenyan Population: The Example of Zinc,” Using Food Science and Technology to Improve Nutrition and Promote National Development 9, no. 1 (2008): 523–535.

[20] F. O. Ibnouf , “The Value of Women's Indigenous Knowledge in Food Processing and Preservation for Achieving Household Food Security in Rural Sudan,” Journal of Food Research 1, no. 1 (2012): 238.

[21] C. Mutungi , H. D. Affognon , A. W. Njoroge , J. Manono , D. Baributsa , and L. L. Murdock , “Triple‐Layer Plastic Bags Protect Dry Common Beans (Phaseolusvulgaris) Against Damage by a Canthoscelidesobtectus (Coleoptera: Chrysomelidae) During Storage,” Journal of Economic Entomology 108, no. 5 (2015): 2479–2488. 26453738 10.1093/jee/tov197

[22] J. Ng'ang'a , C. Mutungi , S. M. Imathiu , and H. Affognon , “Low Permeability Triple‐Layer Plastic Bags Prevent Losses of Maize Caused by Insects in Rural On‐Farm Stores,” Food Security 8, no. 3 (2016): 621–633.

[23] S. R. D. N. Jeeva , R. C. Laloo , and B. P. Mishra (2006). Traditional Agricultural Practices in Meghalaya, NorthEast India.

[24] S. Rawat , “Food Spoilage: Microorganisms and Their Prevention,” Asian journal of Plant science and Research 5, no. 4 (2015): 47–56.

[25] A. Kumar , “Food Preservation: Traditional and Modern Techniques,” Acta Scientific Nutritional Health 3, no. 12 (2019): 45–49.

[26] N. MacCarty , G. Burleson , N. Moses , et al. (2017). “Design and Testing of a High‐Efficiency Rapid Through Put Community‐Scale Water Pasteurization System,” in *International Design Engineering Technical Conferences and Computers and Information in Engineering Conference* Vol. 58134 (American Society of Mechanical Engineers, 2017), V02BT03A016.

[27] F. Melini , V. Melini , F. Luziatelli , and M. Ruzzi , “Raw and Heat‐Treated Milk: From Publichealth Risks to Nutritional Quality,” Beverages 3, no. 4 (2017): 54.

[28] C. Vasanthi , V. Venkataramanujam , and K. Dushyanthan , “Effect of Cooking Temperature and Time on the Physico‐Chemical, Histological and Sensory Properties of Female Carabeef (Buffalo) Meat,” Meatscience 76, no. 2 (2007): 274–280. 10.1016/j.meatsci.2006.11.018 22064296

[29] F. Jabr , “Meat of the Matter,” Scientific American 307, no. 6 (2012): 28–31. 23230793

[30] F. Toldrá , Chapter 9–The Storage and Preservation of Meat: III—Meat Processing. Lawrie's Meat Science (Sawston, United Kingdom: Woodhead Publishing Limited, 2017), 265–296. Eighth Edition.

[31] F. Leroy , A. Geyzen , M. Janssens , L. DeVuyst , and P. Scholliers , “Meat Fermentation at the Crossroads of Innovation and Tradition: A Historical Outlook,” Trends in Food Science & Technology 31, no. 2 (2013): 130–137.

[32] S. J. Santchurn and A. Collignan , “Fermented Poultry Sausages.” in Handbook of Fermented Meat and Poultry, eds. F. Toldrá (Oxford, UK: Blackwell Publishing Ltd, 2007).

[33] C. Verallo and H. Onyeaka , Food Fraud Across World Supply Chain, 2021, https://www.foodsafetyafrica.net/2021/08/19/sector-experts-discuss-on-food-fraud-across-world-supply-chain/.

[34] F. Mustafa and S. Andreescu , “Chemical and Biological Sensors for Food‐Quality Monitoring and Smartpackaging,” Foods 7, no. 10 (2018): 168. 30332833 10.3390/foods7100168 PMC6210272

[35] D. Ghislaine , J. Changwang , and G. Nakweya, Poisons Used to Beautify Food in Africa, 2020, https://www.scidev.net/sub-saharan-africa/features/poisons-used-to-beautify-food-in-africa/.

[36] M. Gagaoua and H. R. Boudechicha , “Ethnic Meat Products of the North African and Mediterranean Countries: An Overview,” Journal of Ethnic Foods 5, no. 2 (2018): 83–98.

[37] H. N. Onyeaka and O. F. Nwabor , “Conventional Preservation and Preservatives,” in Food Preservation and Safety of Natural Products, (Amsterdam, Netherlands: Elsevier, 2022), 51–56.

[38] M. U. Joardder and M. H. Masud , “Challenges and Mistakes in Food Preservation.” Food Preservation in Developing Countries: Challenges and Solutions (Cham: Springer, 2019b), 175–198.

[39] EFSA Panel on Food Additives and Nutrient Sources , “Scientific Opinion on There‐Evaluation of Benzoicacid (E210), Sodiumbenzoate (E211), Potassiumbenzoate (E212) and Calcium Benzoate (E 213) as Food Additives,” EFSAJournal 14, no. 3 (2017): 1–110.

[40] J. D. Piper and P. W. Piper , “Benzoate and Sorbate Salts: A Systematic Review of the Potential Hazards of These Invaluable Preservatives and the Expanding Spectrum of Clinical Uses for Sodium Benzoate,” Comprehensive Reviews in Food Science and Food Safety 16, no. 5 (2017): 868–880. 33371618 10.1111/1541-4337.12284

[41] C. T. DellaValle , Q. Xiao , G. Yang , et al., “Dietary Nitrate and Nitrite Intake and Risk of Colorectal Cancer in the Shanghai Women's Health Study,” International Journal of Cancer 134, no. 12 (2014): 2917–2926. 24242755 10.1002/ijc.28612 PMC3980001

[42] P. Mandal , A. Rai , S. Mishra , A. Tripathi , and M. Das , Mutagenicity: Assays and Applications (Cambridge, MA: AcademicPress, 2018), 133–160. Mutagens in Food.

[43] J. Omojokun , “Regulation and Enforcement of Legislation on Food Safety in Nigeria,” Mycotoxin and Foodsafety in Developing Countries 10 (2013): 251–268.

[44] Z. Ashagrie and D. Abate , “Improvement of Injera Shelf Life Through the Use of Chemical Preservatives,” African Journal of Food, Agriculture, Nutrition and Development 12, no. 5 (2012): 6409–6423.

[45] World Health Organization , (2023 Report 2022: *Pesticide Residues in Food‐Joint FAO/WHO Meeting on Pesticide Residues* (2023).

[46] I. Omwenga , L. Kanja , P. Zomer , J. Louisse , I. M. C. M. Rietjens , and H. Mol , “Organophosphate and Carbamate Pesticide Residues and Accompanying Risks in Commonly Consumed Vegetables in Kenya,” Food Additives & Contaminants: Part B 14, no. 1 (2021): 48–58. 10.1080/19393210.2020.1861661 33353480

[47] E. G. Anaduaka , N. O. Uchendu , R. O. Asomadu , A. L. Ezugwu , E. S. Okeke , and T. P. Chidike Ezeorba , “Widespread Use of Toxic Agrochemicals and Pesticides for Agricultural Products Storage in Africa and Developing Countries: Possible Panacea for Ecotoxicology and Health Implications,” Heliyon 9, no. 4 (2023): e15173. 37113785 10.1016/j.heliyon.2023.e15173 PMC10126862

[48] D. E. Ekundayo (2022). Assessment of Pesticide Effects on Health of Agricultural Workers in Northern Region of Kwara State, Nigeria (Master's thesis, Kwara State University [Nigeria]).

[49] N. Mkhwanazi , C. Adelle , and L. Korsten (2024). Food Safety Governance in South Africa: A Policy Network Approach.

[50] J. K. Adjei , V. Ahormegah , A. K. Boateng , H. K. Megbenu , and S. Owusu , “Fast, Easy, Cheap, Robust and Safe Method of Analysis of Sudan Dyes in Chilli Pepper Powder,” Heliyon 6, no. 10 (2020): e05243. 33088976 10.1016/j.heliyon.2020.e05243 PMC7566101

[51] Y. S. Sulley , L. Quansah , M. Lawal , I. Oboakoh , S. A. Zonu , and D. Yahaya , “Securing the Plate With Forensic Science: Clamping Down on Food Fraud in Ghana,” Current Journal of Applied Science and Technology 42, no. 16 (2023): 1–16.

[52] K. V. Mphaga , D. Moyo , and P. C. Rathebe , “Unlocking Food Safety: A Comprehensive Review of South Africa's Food Control and Safety Landscape From an Environmental Health Perspective,” BMC Public Health 24, no. 1 (2024): 2040. 39080671 10.1186/s12889-024-19589-1 PMC11289970

[53] A. F. Ojonugwa , D. Gwom , and S. Gwom , “The Role and Challenges of the National Agency for Food and Drug Administration and Regulation of Alternative Medicine in Nigeria,” World Health 6 (2021): 52–68.

[54] A. Okoruwa and N. Onuigbo‐Chatta , “Review of Food Safety Policy in Nigeria,” JL Poly and Globalization 110 (2021): 57.

[55] H. Zavala Nacul and C. Revoredo‐Giha , “Food Safety and the Informal Milk Supply Chain in Kenya,” Agriculture & Food Security 11, no. 1 (2022): 8.

[56] C. Kankya , T. Mukungu , J. J. Hoona , et al. (2020). Situation Analysis of Food Safety Control System in Uganda.

[57] African Union , “Side Event on the Africa Food Safety Agency,” in *3rd AU‐EU Agriculture Ministerial Conference, 21 June 2019, Rome, Italy* (2019).

[58] World Health Organization (2020). *The Future of Food Safety: Transforming Knowledge Into Action for People, Economies and the Environment: Technical Summary of the 2019 International Food Safety Conference*s (World Health Organization).

[59] H. Onyeaka , M. Ukwuru , C. Anumudu , and A. Anyogu , “Food Fraud in Insecure Times: Challenges and Opportunities for Reducing Food Fraud in Africa,” Trends in Food Science & Technology 125 (2022): 26–32.

[60] H. Onyeaka , S. Ghosh , K. Obileke , et al. (2022). *Chemical Toxicants in Food: Improvement and Sustainability of Best Practices*. Available at SSRN4138242.

[61] N. Nwuneli (2018). *Fake Processed Food Is Becoming an Epidemic in African Urbanlife*, https://qz.com/africa/1226112/fake-food-or-fraud-food-in-nigeria-kenya-and-other-african-countries/.

[62] S. M. VanRuth and O. Nillesen , “Which Company Characteristics Make a Food Business at Risk for Food Fraud?,” Foods 10, no. 4 (2021): 842. 33924386 10.3390/foods10040842 PMC8069500

[63] I. C. Shaw , “Chemical Residues, Food Additives and Natural Toxicants in Food–Thecocktail Effect,” International Journal of Food Science & Technology 49, no. 10 (2014): 2149–2157.

[64] H. Onyeaka , M. S. Kalane , A. T. Guta , and P. Tamasiga , “Food Fraud Amid COVID‐19 in Sub‐Saharan Africa,” Public Health in Practice (Oxford, England) 3 (2022): 100234. 36101763 10.1016/j.puhip.2022.100234 PMC9461351

[65] A. T. Gemechu , Y. B. Tola , T. K. Dejenie , D. R. Grace , F. B. Aleka , and T. T. Ejeta , “Assessment of Butter Adulteration Practices and Associated Food Safety Issuesalong the Supply Chain in Traditional Communities in the Central Highlands and South West Mid Lands of Ethiopia,” Journal of Food Protection 84, no. 5 (2021): 885–895. 33320941 10.4315/JFP-20-355

[66] M. Mburu , C. Komu , O. Paquet‐Durand , B. Hitzmann , and V. Zettel , “Chia Oil Adulteration Detection Based on Spectroscopic Measurements,” Foods 10, no. 8 (2021): 1798. 34441575 10.3390/foods10081798 PMC8392156

[67] K. Edwards , M. Manley , L. C. Hoffman , and P. J. Williams , “Non‐Destructive Spectroscopic and Imaging Techniques for the Detection of Processed Meat Fraud,” Foods 10, no. 2 (2021): 448. 33670564 10.3390/foods10020448 PMC7922372

[68] E. Teye (2022). *Tackling the Other Pandemic: Food Fraud*, https://theanalyticalscientist.com/business-education/tackling-the-other-pandemic-food-fraud.

[69] P. K. Singh , R. P. Singh , P. Singh , and R. L. Singh , Food Safety and Human Health (Cambridge, MA: AcademicPress, 2019), 15–65. Food Hazards: Physical, Chemical, and Biological.

[70] I. Rasooli , “Food Preservation–A Biopreservative Approach,” Food 1, no. 2 (2007): 111–136.

[71] C. F. S. Da Araújo , M. V. Lopes , M. R. Vasquez , et al., “Cadmium and Lead in Seafood From the Aratubay, Brazil and the Humanhealth Risk Assessment,” Environmental Monitoring and Assessment 188, no. 4 (2016): 1–12. 27359001 10.1007/s10661-016-5262-y

[72] I. A. Rather , W. Y. Koh , W. K. Paek , and J. Lim , “The Sources of Chemical Contaminants in Food and Their Health Implications,” Frontiers in Pharmacology 8 (2017): 830. 29204118 10.3389/fphar.2017.00830 PMC5699236

[73] L. A. Thompson and W. S. Darwish , “Environmental Chemical Contaminants in Food: Review of a Global Problem,” Journal of Toxicology 2019, no. 1 (2019): 2345283. 30693025 10.1155/2019/2345283 PMC6332928

[74] J. Spink and D. C. Moyer , “Defining the Public Health Threat of Food Fraud,” Journal of Food Science 76, no. 9 (2011): R157–R163. 22416717 10.1111/j.1750-3841.2011.02417.x

[75] A. J. Alldrick , Hand book of Hygiene Controlin the Food Industry (Sawston, UK: Woodhead Publishing, 2016), 89–102, 10.1016/b978-0-08-100155-4.00007-8. Chemical Hazards.

[76] C. J. Carter and R. A. Blizard , “Autism Genes Are Selectively Targeted by Environmental Pollutants Including Pesticides, Heavy Metals, Bisphenol A, Phthalates and Many Others in Food, Cosmetics or Household Products,” Neuro Chemistry International 101 (2016): 83–109. 10.1016/j.neuint.2016.10.011 27984170

[77] R. H. Stadler and D. R. Lineback , “Process‐Induced Food Toxicants.” Occurrance, Formation, Mitigation and Health Risks (New Jersey: A John Wiley and Sons, Inc., Publication. Hoboken, 2009).

[78] W. Zhang and J. Xue , “Economically Motivated Food Fraud and Adulteration in China: Ananalysis Based on 1553 Media Reports,” Food Control 67 (2016): 192–198.

[79] C. Jia and D. Jukes , “The National Food Safety Control System of China–A Systematic Review,” Food Control 32, no. 1 (2013): 236–245.

[80] K. Everstine , J. Spink , and S. Kennedy , “Economically Motivated Adulteration (EMA) of Food:Common Characteristics of EMA Incidents,” Journal of Food Protection 76, no. 4 (2013): 723–735. 23575142 10.4315/0362-028X.JFP-12-399

[81] I. H. Caspersen , M. Haugen , S. Schjølberg , et al., “Maternal Dietary Exposure to Dioxins and Polychlorinated Biphenyls (PCBS) is Associated With Language Delay in 3 year Old Norwegian Children,” Environment International 91 (2016): 180–187. 26970589 10.1016/j.envint.2016.02.031

[82] L. A. Thompson , Y. Ikenaka , Y. B. Yohannes , et al., “Human Health Risk From Consumption of Marine Fish Contaminated With DDT and Its Metabolites in Maputo Bay, Mozambique,” Bulletin of Environmental Contamination and Toxicology 100, no. 5 (2018): 672–676. 29546500 10.1007/s00128-018-2323-7

[83] A. Carocci , A. Catalano , G. Lauria , M. S. Sinicropi , and G. Genchi , “16 A Review on Mercury Toxicity Infood,” Food Toxicology 2 (2016): 315.

[84] M. O. Gribble , A. Cheng , R. D. Berger , L. Rosman , and E. Guallar , “Mercury Exposure and Heart Rate Variability: A Systematic Review,” Current Environmental Health Reports 2, no. 3 (2015): 304–314. 26231507 10.1007/s40572-015-0053-0 PMC4800007

[85] B. Fernandes Azevedo , L. Barros Furieri , F. M. Peçanha , et al., “Toxic Effects of Mercury on the Cardiovascular and Central Nervous Systems,” Journal of Biomedicine and Biotechnology 2012 (2012): 1–11, 10.1155/2012/949048. 22811600 PMC3395437

[86] G. Ginsberg , B. Sonawane , R. Nath , and P. Lewandowski , “Methylmercury‐Induced Inhibition of Paraoxonase‐1 (PON1)—Implications for Cardiovascular Risk,” Journal of Toxicology and Environmental Health, Part A 77, no. 17 (2014): 1004–1023. 25072822 10.1080/15287394.2014.919837

[87] D. A. Rizzetti , J. G. D. Torres , A. G. Escobar , et al., “Apocynin Prevents Vascular Effects Caused by Chronic Exposure to Low Concentrations of Mercury,” PLoS One 8, no. 2 (2013): e55806. 23390552 10.1371/journal.pone.0055806 PMC3563583

[88] G. Genchi , M. Sinicropi , A. Carocci , G. Lauria , and A. Catalano , “Mercury Exposure and Heart Diseases,” International Journal of Environmental Research and Public Health 14, no. 1 (2017): 74. 28085104 10.3390/ijerph14010074 PMC5295325

[89] G. R. Sampaio , G. M. Guizellini , S. A. da Silva , et al., “Polycyclic Aromatic Hydrocarbons in Foods: Biological Effects, Legislation, Occurrence, Analytical Methods, and Strategies to Reduce Their Formation,” International Journal of MolecularSciences 22, no. 11 (2021): 6010. 10.3390/ijms22116010 PMC8199595 34199457

[90] X. Duan , G. Shen , H. Yang , et al., “Dietaryintake Polycyclic Aromatic Hydrocarbons (PAHs) and Associated Cancer Risk in a Cohort of Chinese Urban Adults: Inter‐and Intra‐Individual Variability,” Chemosphere 144 (2016): 2469–2475. 26619312 10.1016/j.chemosphere.2015.11.019 PMC4695288

[91] L. R. Johnson , Physiology of the Gastrointestinal Tract (Amsterdam, The Netherlands: Elsevier, 2006).

[92] K. H. Kim , S. A. Jahan , E. Kabir , and R. J. Brown , “A Review of Airborne Polycyclic Aromatichydrocarbons (PAHs) and Their Human Healtheffects,” Environment International 60 (2013): 71–80. 24013021 10.1016/j.envint.2013.07.019

[93] Z. Zelinkova and T. Wenzl , “The Occurrence of 16EPAPAHS in Food–A Review,” Polycyclic Aromatic Compounds 35, no. 2–4 (2015): 248–284. 26681897 10.1080/10406638.2014.918550 PMC4673601

[94] O. C. Ifegwu and C. Anyakora , Polycyclic Aromatic Hydrocarbons: Part I. Exposure and Advances in Clinical Chemistry (Amsterdam, The Netherlands: Elsevier, 2015) Volume 72. 1st ed., 277–304. 26471085 10.1016/bs.acc.2015.08.001

[95] B. W. Katona and J. P. Lynch , Mechanisms of Gastrointestinal Malignancies. Physiology of the Gastrointestinal Tract (Amsterdam, The Netherlands: Elsevier, 2018), 1615–1642.

[96] S. Choudhuri , G. W. Patton , R. F. Chanderbhan , A. Mattia , and C. D. Klaassen , “From Classical Toxicology to Tox21: Some Critical Conceptual and Technological Advances in the Molecular Understanding of the Toxic Response Beginning From the Last Quarter of the 20th Century,” Toxicological Sciences 161, no. 1 (2018): 5–22. 28973688 10.1093/toxsci/kfx186 PMC5837539

[97] I. M. Ezeonu , C. M. Ononugbo , and A. C. Ike , “Molecular Characterization of Listeriamonocytogenes Isolated From a Ready‐To‐Eat Fermented Milk and Cereal Product, Fura‐De‐Nunu,” African Journal of Microbiology Research 12, no. 19 (2018): 448–455.

[98] S. Sciuto , G. Esposito , L. Dell'Atti , C. Guglielmetti , P. L. Acutis , and F. Martucci , “Rapid Screening Technique to Identify Sudan Dyes (ItoIV) in Adulterated Tomato Sauce, Chillipowder, and Palmoil by Innovative High Resolution Mass Spectrometry,” Journalof Food Protection 80, no. 4 (2017): 640–644. 10.4315/0362-028X.JFP-16-313 28294682

[99] S. Yao‐Say solomon adade , H. Lin , H. Jiang , et al., “Fraud Detection in Crude Palm Oil Using SERS Combined With Chemometrics,” Food Chemistry 388 (2022): 132973. 35447589 10.1016/j.foodchem.2022.132973

[100] E. Teye , C. L. Amuah , T. McGrath , and C. Elliott , “Innovative and Rapid Analysis for Rice Authenticity Using Hand‐Held NIR Spectrometry and Chemometrics,” Spectrochimica ActaPart A: Molecular and Biomolecular Spectroscopy 217 (2019a): 147–154. 10.1016/j.saa.2019.03.085 30933778

[101] E. Teye , C. Elliott , L. K. Sam‐Amoah , and C. Mingle , “Rapid and Nondestructive Fraud Detection of Palm Oil Adulteration With Sudan Dyes Using Portable NIR Spectroscopic Techniques,” Food Additives and Contaminants: Part A 36, no. 11 (2019b): 1589–1596. 10.1080/19440049.2019.1658905 31535956

[102] E. K. Anyidoho , E. Teye , and R. Agbemafle , “Differentiation of Organic CocoaBeans and Conventional Ones by Using Handheld NIR Spectroscopy and Multivariate Classification Techniques,” International Journal of Food Science 2021 (2021): 1–13, 10.1155/2021/1844675. PMC8627362 34845434

[103] K. Edwards , M. Manley , L. C. Hoffman , et al., “Differentiation of South African Game Meat Using Near‐Infrared (NIR) Spectroscopy and Hierarchical Modelling,” Molecules 25, no. 8 (2020): 1845. 32316308 10.3390/molecules25081845 PMC7221759

[104] K. K. Ejeahalaka , P. Mclaughlin , and S. L. W. On , “Monitoring the Composition, Authenticity and Quality Dynamics of Commercially Available Nigerian Fat‐Filled Milkpowders Under Inclement Conditions Using NIRs, Chemometrics, Packaging and Microbiological Parameters,” Food Chemistry 339 (2021): 127844. 32829243 10.1016/j.foodchem.2020.127844

[105] T. Kuballa , T. Hausler , A. O. Okaru , et al., “Detection of Counterfeit Brand Spirits Using 1H NMR Fingerprints in Comparisonto Sensory Analysis,” Food Chemistry 245 (2018): 112–118. 29287330 10.1016/j.foodchem.2017.10.065

[106] R. Consonni , L. R. Cagliani , and C. Cogliati , “NMR Based Geographical Characterization of Roasted Coffee,” Talanta 88 (2012): 420–426. 22265520 10.1016/j.talanta.2011.11.010

[107] M. Worku , H. R. Upadhayay , K. Latruwe , et al., “Differentiating the Geographical Origin of Ethiopian Coffee Using XRF‐ and ICP‐Based Multi‐Element and Stable Isotope Profiling,” FoodChemistry 290 (2019): 295–307. 10.1016/j.foodchem.2019.03.135 31000050

[108] N. P. Kalogiouri , R. Aalizadeh , M. E. Dasenaki , and N. S. Thomaidis , “Authentication of Greek PDO Kalamata Table Olives: A Novel Non‐Target High Resolution Mass Spectrometric Approach,” Molecules 25, no. 12 (2020): 2919. 32599950 10.3390/molecules25122919 PMC7355929

[109] P. Bertrand , H. Ahmed , R. Ngwafor , and C. Frazzoli , “Toxicovigilance Systems and Practices in Africa,” Toxics 4, no. 3 (2016): 13. 29051419 10.3390/toxics4030013 PMC5606664

[110] T. Yahaya , A. Ukeoma , M. Musa , L. Abdullahi , A. Muhammad , and E. John , “Demographics and Chemical Preservatives Used by Vegetable and Fruit Retailers Selected Across Markets in Lagos, Southwestern Nigeria,” Tropical Environment, Biology, and Technology 1, no. 2 (2023): 76–85.

[111] R. Kilonzo and M. Gathura (2019). The Kenya Food Control System. Ukwueze, F. O. (2019). Evaluation of Food Safety and Quality Regulations in Nigeria. JL Poly and Globalization, 92, 148, https://www.fao.org/fileadmin/user_upload/agns/news_events/Pre_CCAFRICA_KenyaEN.pdf.

[112] V. K. Cheruiyot , “The Challenges of Managing Stakeholder Based Influence on Service Delivery in Standards Regulatory Agencies, a Case Study of Kenya Bureau of Standards,” Strategic Journal of Business & Change Management 3, no. 4 (2016): 75.

[113] S. Ohene‐Darko , “Food Safety Governance in the Cape Coast Metropolis, Ghana” (Doctoral dissertation, University of Cape Coast, 2018).

[114] W. Birke and F. Zawide , “Transforming Research Results in Food Safety to Community Actions: A Call for Action to Advance Food Safety in Ethiopia,” Environment and Ecology Research 7, no. 3 (2019): 153–170.

[115] M. Lavanya , S. K. R. Namasivayam , and A. John , “Developmental Formulation Principles of Food Preservatives by Nanoencapsulation—Fundamentals, Application, and Challenges,” Applied Biochemistry and Biotechnology 196 (2024): 7503–7533. 38713338 10.1007/s12010-024-04943-1

[116] S. Priyanka , R. S. Arvind Bharani , and A. John , “Biocompatible Green Technology Principles for the Fabrication of Food Packaging Material With Noteworthy Mechanical and Antimicrobial Properties–A Sustainable Developmental Goal Towards the Effective, Safe Food Preservation Strategy,” Chemosphere 336 (2023): 139240. 37348611 10.1016/j.chemosphere.2023.139240

[117] S. Priyanka , S. K. R. Namasivayam , S. Sudha , M. Lavanya , and T. Abiraamavalli , “Potential Biological Active Bacteriocin Production by Bifidobacterium via Eco‐Friendly, Low‐Cost Solid State Fermentation Principle,” Environmental Quality Management 34, no. 1 (2024): 1–8, 10.1002/tqem.22210.

[118] S. K. R. Namasivayam , A. John , R. S. Arvind Bharani , M. Kavisri , and M. Moovendhan , “Biocompatible Formulation of Cationic Antimicrobial Peptide Polylysine (PL) Through Nanotechnology Principles and Its Potential Role in Food Preservation—A Review,” International Journal of Biological Macromolecules 222 (2022): 1734–1746. 36206840 10.1016/j.ijbiomac.2022.09.238

[119] S. K. R. Namasivayam , M. Manohar , J. A. Kumar , et al., “Green Chemistry Principles for the Synthesis of Anti Fungal Active Gum Acacia‐Gold Nanocomposite‐Natamycin (Ga‐AuNC–NT) Against Food Spoilage Fungal Strain Aspergillus Ochraceopealiformis and Its Marked Congo Red Dye Adsorption Efficacy,” Environmental Research 212 (2022): 113386. 35569536 10.1016/j.envres.2022.113386

